# Phytoplankton–Bacteria Interactions 1.0

**DOI:** 10.3390/microorganisms11051188

**Published:** 2023-05-01

**Authors:** Katherina Petrou

**Affiliations:** School of Life Sciences, University of Technology Sydney, Sydney, NSW 2007, Australia; katherina.petrou@uts.edu.au

Phytoplankton and bacteria regulate many essential functions in aquatic ecosystems. As the primary producers and primary degraders of the aquatic environment, they form the top and tail of the food web, and like alchemists working in concert, they are responsible for producing, transferring and transforming chemicals. Various aspects of these important ecological functions are often explored separately, with the two groups (bacteria and phytoplankton) playing their respective roles and working independently from each other. Yet, the more we uncover about phytoplankton–bacterial relationships, the greater the realization that in many cases, it is the intricate interactions between these two microbial groups that dominate many of these processes. Current research is revealing that although they belong to different domains of life, interactions between phytoplankton and bacteria are ubiquitous in the aquatic environment, and whether obligate, facultative, mutualistic or parasitic, these inter-domain interactions play an important role in aquatic ecosystems.

This Special Issue focuses on the importance of phytoplankton–bacteria relationships in aquatic environments, highlighting new work and scientific advances in our understanding of the intricacies of such interactions. In this issue, through a collection of papers, we look at diverse forms of chemically mediated interactions at different scales and explore phytoplankton–bacterial associations in both laboratory cultures and natural ocean systems.

Phytoplankton–bacteria relationships are often facilitated directly by cell-to-cell attachment or by association and proximity, and many studies have demonstrated growth benefits which have been directly derived from bacterial co-culturing. Co-benefits such as the exchange of metabolic compounds, including vitamin B12, between the two organisms have been identified for some microalgal strains [1]; the bacteria provide B12 to the algae in exchange for carbon substrate for growth from the algal exudates. Recent work has been shown that the cross-exchange of B-vitamins underpins a mutualistic interaction between *Ostreococcus tauri* and the alpha-proteobacterium *Dinoroseobacter shiba* [2]. Using *O. tauri* with a new bacterial isolate, *Marinobacter algicola,* Pinto et al. [3] investigated the dependency and nature of the association between *O. tauri* and *M. algicola*. They employed a dual-approach of co-culturing experiments and genomic information to look at fitness in relation to metabolite exchange and quorum sensing capacity. Contrary to their expectations, they found no advantage of co-culturing conferred to *O. tauri* and no genomic evidence of quorum sensing in *M. algicola*, and suggested that the persistence of *M. algicola* in *O. tauri* cultures, along with other cultures, is likely a result of opportunism.

Quorum sensing (QS), a mechanism by which bacteria sense cell density of their own species, is a well-known process that facilitates coordinated gene expression and physiology across an entire bacterial community. More recent research is focusing on how QS signaling is being used inter-specifically and across domains. Dow [4] presented a review of the current state of knowledge of QS-mediated algae–bacteria interactions, summarizing the role of bacterially derived QS compounds in microalgal fitness and how microalgae, in turn, may regulate their influence.

In addition to close relationships and direct associations, interactions can also occur indirectly via the release of a suite of chemical compounds and metabolites into the surrounding water, collectively referred to as dissolved organic carbon (DOC). The large reservoir of DOC forms the primary source of substrates for heterotrophic bacteria. In particular, research to date has found that the marine bacterial assemblages associated with phytoplankton are consistently dominated by Rhodobacterales, Flavobacteriales and Gammaproteobacteria. Similar associations are described in the paper by Le Reun et al. [5]. Using amplicon sequencing data obtained from an Australia-wide network of oceanographic sites, they explored the seasonal and biogeographical patterns of diatom communities from coastlines around Australia and found that, along with nutrients and temperature, specific groups of bacteria previously implicated in mutualistic associations with diatoms explained the spatial dynamics of diatom communities. They also found significant co-occurrence between specific bacterial groups (Roseobacter and Flavobacteria) and the diatom genera *Skeletonema*, *Thalassiosira* and *Cylindrotheca*, which may represent ecologically important interactions between diatoms and bacteria in coastal waters.

When metabolites are concentrated in proximity to a phytoplankton cell, the resulting cloud of a carbon pool is referred to as the phycosphere. The phycosphere provides a concentration gradient for proximate motile bacteria to swim toward the cell and access the compounds released by the algal cell. Close associations with particulate and dissolved organic carbon were explored in two papers within this issue, with a focus on the phytoplankton-derived sulfur compound dimethylsulfoniopropionate (DMSP) and exopolymeric substances (EPS) in complexation with iron (Fe). Phytoplankton and bacteria are intertwined in the regulation of the marine sulfur cycle. In its simplest portrayal, the production of DMSP by phytoplankton and its conversion to DMS by bacteria has long been touted as a biologically mediated mode of climate regulation. The paper by Fernandez et al. [6] in this Special Issue looked at a relatively new aspect of DMSP cycling by analyzing the uptake by the community from open water- and coral reef-associated pelagic microbial communities. Rather than production and conversion, they considered uptake and accumulation within different size fractions of the marine microbial community. Their study revealed active uptake of DMSP in all size fractions, with the largest cells forming the major DMSP sink. They also found that the microbial responses differed depending on the communities’ carbon and sulfur requirements. Inner reef communities showed a preference towards cleaving DMSP into the climatically active aerosol dimethyl sulfide (DMS), whereas outer reef communities appeared to be limited in sulfur and carbon, thereby preferring available DMSP to be utilized by the cells. In contrast to DMSP, the role of marine bacteria in regulating iron availability for phytoplankton is largely unknown. It is well established that organic ligands, including exopolymeric substances (EPS), form complexes with iron (Fe) and can regulate the growth of phytoplankton, particularly in iron-limited regions of the ocean. Blanco-Ameijeras et al. [7] investigated whether these ligand-Fe complexes also influence the abundance and diversity of bacteria and viruses. Their study revealed a decoupling between Fe-stimulated phytoplankton growth and EPS-modulated prokaryotic and viral community dynamics, with EPS forming an important carbon source that is capable of enhancing the microbial loop.

The final paper in this Special Issue considers the role of microbes in shaping physiologies in response to environmental stress. Microbial communities are susceptible to changes in environmental conditions, and close associations between microalgae and bacteria can be used to assess stress, as shifts in their abundance and diversity can indicate changes in the physiological condition. Jiayuan Liang et al. [8] provided insight into the regulation of coral microbial populations in response to warming events. Their detailed analyses on community dynamics and physiology demonstrated changes in both bacterial and algal symbiont dominance both with the onset of warming and after heat stress. The authors proposed the post-warming changes in the bacterial consortia could form a possible indicator of recovery in the coral holobiont.

Together, phytoplankton and bacteria are principal players in modulating biogeochemistry and nutrient cycling; by way of their effect on each other’s physiology and metabolism, they often define the productivity of ecosystems. Therefore, examining processes that govern phytoplankton–bacterial networks and associations is important if we are to form a deeper understanding of the underpinnings of our aquatic ecosystems.
